# Supplementary material for: Does green innovation moderate between FDI and environmental sustainability? Empirical evidence from South Asia
Source: PLoS One. 2023 Nov 9;18(11):e0288532. doi: 10.1371/journal.pone.0288532 (PMC10635563; doi:10.1371/journal.pone.0288532)
Supplement: Attachment — Submitted filename: PONE_review.docx [file pone.0288532.s001.docx]

I thoroughly read the research paper entitled “Does Green Innovation Moderate between FDI and Environmental Sustainability? Empirical Evidence from South Asia”. The topic is interesting and will contributes to Environmental Economic literature. Before to publish the paper, revise the paper according to my comments and suggestions.

1. Please clearly elaborate the novelty of your research paper in the introduction section.
2. Please briefly clarify your methodology in the introduction section
3. Please briefly discuss your results at the end of introduction section. So that readers be convenient while read your research paper.
4. The objectives should not be in bulletin. Its should be in paragraph. Its not thesis.
5. The very current literature is missing on Environmental sustainability and FDI in south Asia and global. Please update the literature. For example,

https://doi.org/10.1016/j.apenergy.2023.120836,

https://doi.org/10.1016/j.renene.2023.02.035

https://doi.org/10.1016/j.ribaf.2019.101129

https://doi.org/10.1108/IJOEM-03-2022-0395

10.1061/(ASCE)IS.1943-555X.0000724

DOI 10.1108/JES-03-2020-0123

Good luck!
