# Supplementary material for: Does green innovation moderate between FDI and environmental sustainability? Empirical evidence from South Asia
Source: PLoS One. 2023 Nov 9;18(11):e0288532. doi: 10.1371/journal.pone.0288532 (PMC10635563; doi:10.1371/journal.pone.0288532)
Supplement: Attachment — Submitted filename: Reviewers response.docx [file pone.0288532.s002.docx]

Review and Response Sheet

We are very grateful to respected editor and reviewers for their time and efforts to provide the valuable insights for the improvement of this paper. We have revised the manuscript under the suggestions of respected reviewers. The changes are highlighted with yellow color in each part of the manuscript the response of each comment is given below.

**Reviewer #1**

| Comments | Author Response |
| --- | --- |
| There is no research question or policy relevance. | The author has added the research questions. |
| The literature review pinpoints no research gap. | The study highlighted the research gap |
| The empirical model is flawed. It suffers from endogeneity bias (reverse causality and omitted variable). | Authors has applied the causality test and the for the endogeneity problem authors have employed FMOLS and DOLS estimation. |
| Results are not discussed. They are only reported. | Results are discussed in detail. |
| There are no implications drawn from the study. | The author draws Policy implications from the study. |
| The language is below publication standard. | The paper is now proofread by a native speaker. |

**Reviewer 2**

| Comments | Author Response |
| --- | --- |
| The manuscript is well written. The topic is very interesting. However, authors need to revise the abstract and make it clearer and crisper. | Thank you for your comment. Author has revised the abstract make it clearer and more understandable. |
| Add value addition in the introduction, you may read the suggested papers and get the idea how to write down the value addition. | Author added the value addition in the introduction and revised the whole intro. |
| Please add these papers in literature review or introduction to make it more rigorous and updated:  1. https://link.springer.com/article/10.1007/s11356-022-21401-9  2.https://link.springer.com/article/10.1007/s11356-022-21339-y  3.https://link.springer.com/article/10.1007/s11356-022-22587-8  4.https://www.sciencedirect.com/science/article/abs/pii/S  0301420722003579  5.https://link.springer.com/article/10.1007/s11356-022-23355-4 | Thank you for suggesting the valuable papers which increased the empirical evidence and justification. Author has cited the all relevant papers. |
| Review the English language and correct grammar and spelling errors. | The paper is now proofread by native speaker. |

**Reviewer #3**

| Comments | Author Response |
| --- | --- |
| Please clearly elaborate the novelty of your research paper in the introduction section. | Thank you for pointing out the important point. The author has highlighted the contribution of the study. |
| Please briefly clarify your methodology in the introduction section | The author has clarified the methodology in the introduction section now it is clearer and more understandable. |
| Please briefly discuss your results at the end of introduction section. So that readers be convenient while read your research paper. | The author has added the results at the end of the introduction section. |
| The objectives should not be in bulletin. Its should be in paragraph. Its not thesis. | The author has rewritten the objectives according to suggestions. |
| The very current literature is missing on Environmental sustainability and FDI in south Asia and global. Please update the literature. For example,  https://doi.org/10.1016/j.apenergy.2023.120836,  https://doi.org/10.1016/j.renene.2023.02.035  https://doi.org/10.1016/j.ribaf.2019.101129  https://doi.org/10.1108/IJOEM-03-2022-0395  10.1061/(ASCE)IS.1943-555X.0000724  DOI 10.1108/JES-03-2020-0123 | Thank you for suggesting the valuable recent papers which increased the empirical evidence and justification. The author has cited all relevant papers. |

Thank you,

Sincerely
